# *N*,*N'*-(Hexane-1,6-diyl)bis(4-methyl-*N*-(oxiran-2-ylmethyl)benzenesulfonamide): Synthesis via cyclodextrin mediated *N*-alkylation in aqueous solution and further Prilezhaev epoxidation

**DOI:** 10.3762/bjoc.9.318

**Published:** 2013-12-09

**Authors:** Julian Fischer, Simon Millan, Helmut Ritter

**Affiliations:** 1 Institute of Organic Chemistry and Macromolecular Chemistry, Heinrich-Heine-University Düsseldorf, Universitätsstraße 1, D-40225 Düsseldorf, Germany

**Keywords:** alkylation, cyclodextrins, epoxy, rheology, sulfonamides

## Abstract

*N*-alkylation of *N*,*N'*-(hexane-1,6-diyl)bis(4-methylbenzenesulfonamide) with allyl bromide and subsequent Prilezhaev reaction with *m*-chloroperbenzoic acid to give *N*,*N'*-(hexane-1,6-diyl)bis(4-methyl-*N*-(oxiran-2-ylmethyl)benzenesulfonamide) is described. This twofold alkylation was performed in aqueous solution, whereby α-, and randomly methylated β-cyclodextrin were used as adequate phase transfer catalysts and the cyclodextrin–guest complexes were characterized by ^1^H NMR and 2D NMR ROESY spectroscopy. Finally, the curing properties of the diepoxide with lysine-based α-amino-ε-caprolactam were analyzed by rheological measurements.

## Introduction

Various diepoxides easily react with amines or diamines to form cross-linked, cyclic or linear addition-polymers, which are implemented in construction, electronic, aerospace, medical and dental industries [1–2]. Hereby, bisphenol A diglycidyl ether (BADGE) is often used [3–4]. However, non-bisphenol A based diepoxides are subject to intensive research [5–9]. The industrial synthesis of BADGE and other commercially available diepoxides proceeds mainly by using epichlorohydrin, whereby usually several side products and oligomers are formed in significant concentrations [10–12]. An alternative route is a two-step reaction via *N*-allylation and further Prilezhaev epoxidation with peroxides [13–15]. The solubility of hydrophobic reactants in water can be increased significantly by cyclodextrins (CD) and thereby the use of organic solvents can be reduced [16–18]. To our best knowledge, CD mediated *N*-alkylation of sulfonamides is not yet described. Generally, only a few examples are known in literature about CD assisted alkylation of amines in aqueous solution [19–22]. Hence, in this work, we wish to present our direct and CD mediated method to alternative sulfonamide based diepoxides, focusing on one characteristic example. Furthermore, the polymerization of these types of diepoxides was investigated with lysine-based α-amino-ε-caprolactam through rheological measurements.

## Results and Discussion

**Scheme 1:**
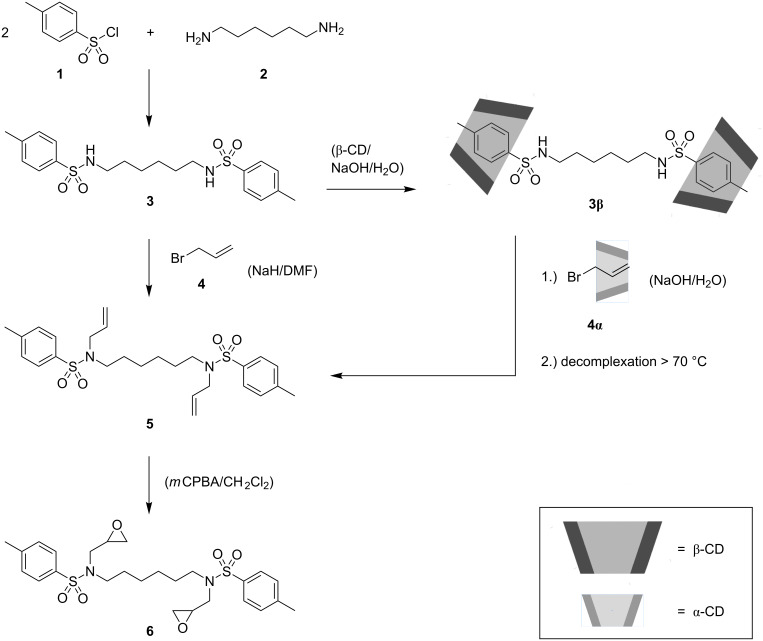
Synthesis of sulfonamide **3**, *N*-alkylation of **3** in organic solution and of CD-complex (**3β**) in aqueous phase to obtain **5** and the subsequent epoxidation with *m*-chloroperbenzoic acid (*m*CPBA) to yield product **6**.

*N*,*N'*-(Hexane-1,6-diyl)bis(4-methylbenzenesulfonamide) (**3**), as precursor for the subsequent *N*-alkylation and further Prilezhaev epoxidation, was synthesized easily from *p*-toluenesulfonyl chloride (**1**) and hexamethylenediamine (**2**) [23]. The resulting crystalline sulfonamide was first described in 1927 prepared from 1,6-dibromohexane and *p*-toluenesulfonamide [24]. The subsequent two-fold *N*-alkylation of **3** with allyl bromide (**4**) was conducted in *N*,*N*-dimethylformamide as solvent, as well as in aqueous solution via CD-complexation (Scheme 1).

**Figure 1:**
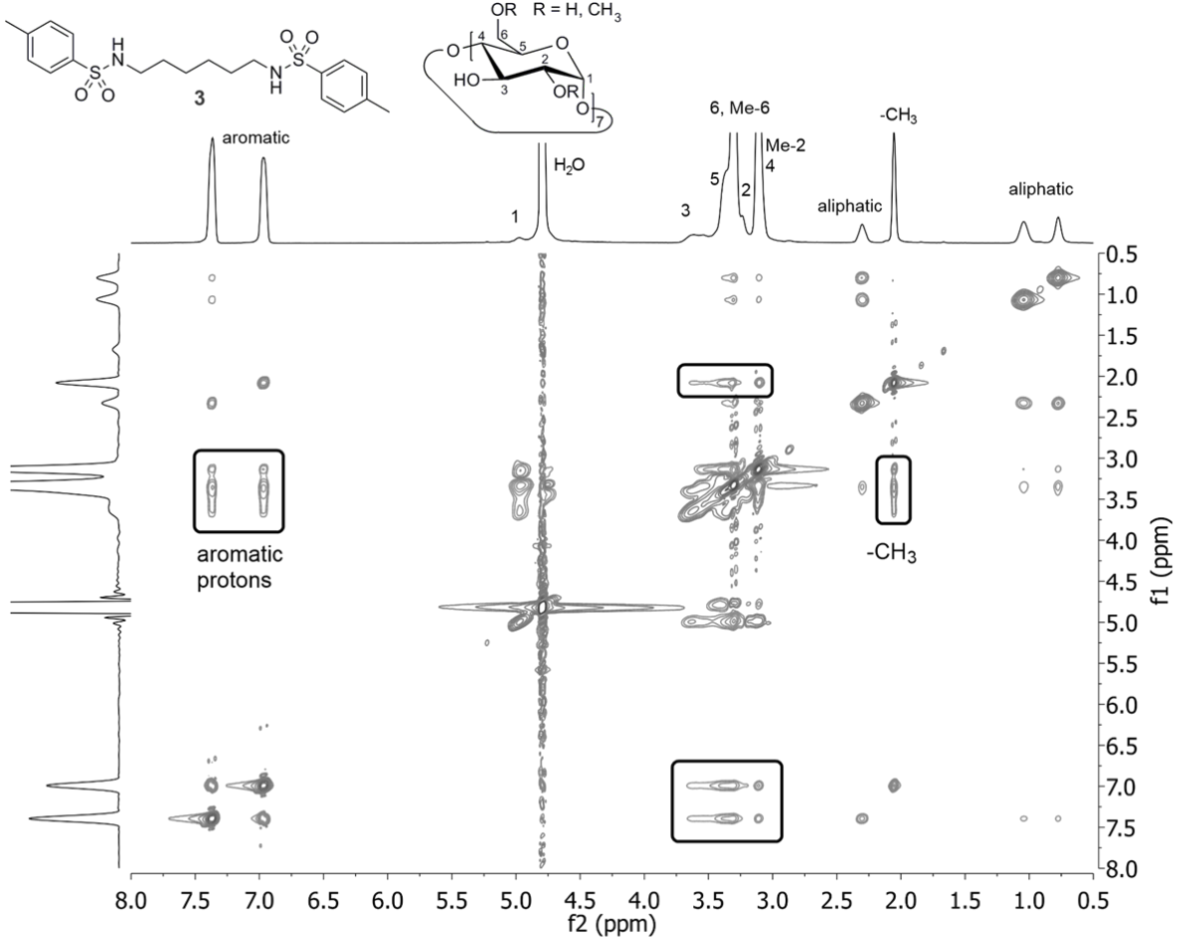
2D NMR ROESY spectrum of the complex of **3** with β-CD in D_2_O, displaying the interaction of the tosyl protons with the β-CD protons.

Unmodified **3** is neither in a neutral nor in a basic milieu significantly soluble. However, by complexation of **3** with two equivalents of randomly methylated β-cyclodextrin (β-CD) to give **3β**, water solubility could be increased distinctively. Characterization of the inclusion complex of β-CD with **3** in D_2_O solution was conducted using 2D NMR ROESY spectroscopy (Figure 1).

Thereby, proton signals at 7.0 and 7.4 ppm can be assigned to the aromatic protons of **3**. Furthermore, the protons of its *p*-methyl moiety demonstrate a singlet signal at 2.1 ppm. As illustrated in Figure 1, interaction of these protons with the inner protons of β-CD in the range of 3.3 to 3.8 ppm is visible. This strongly indicates a self-agglomeration of the tosyl groups of **3** with β-CD. Further confirmation of these findings is a perceivable downfield shifting of the proton signals of **3β** compared to **3** suspended in D_2_O, since they are magnetically shielded by the β-CD cavity.

Additionally, the water solubility of **4** is limited as well (3.83 g L^−1^) [25]. Since the stability of CD–guest complexes often depends on the size of the guest molecule relative to the CD cavity, stability constants of unbranched alkyl chains or vinyl groups are the highest with α-CD (six glucose units) and for tosyl groups with β-CD (seven glucose units), respectively [18]. Therefore, the water solubility of **4** was increased by addition of α-CD (**4α**). 2D NMR ROESY spectroscopy was also performed for **4α**. As expected, the protons of the allyl-group at 5.9 ppm, 5.1 ppm and 4.0 ppm correlate with the α-CD protons in the range of 3.9 to 3.5 ppm (Figure S1, Supporting Information File 1).

**Scheme 2:**
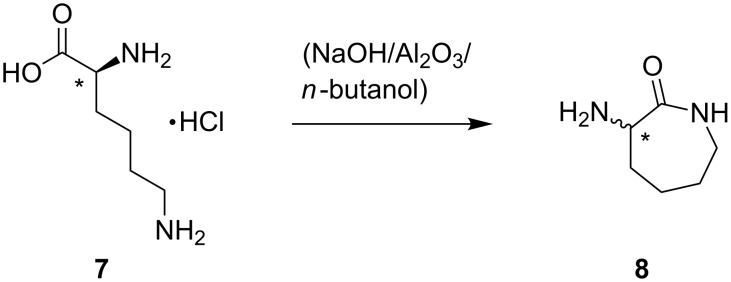
Cyclization of L-(+)-lysine monohydrochloride to give racemate **8**.

Two-fold *N*-alkylation of β-CD-complexed *N*,*N'*-(hexane-1,6-diyl)bis(4-methylbenzenesulfonamide) (**3β**) with **4α** in aqueous solution gave *N*,*N'*-(hexane-1,6-diyl)bis(*N*-allyl-4-methylbenzenesulfonamide) (**5**), which could easily be precipitated and separated from the CD-complex by heating, since decomplexation occurred over 70 °C. The remaining aqueous CD-solution can be reused in principle, since in an alkaline milieu CD-rings are stable. A comparison of the ^1^H NMR spectra of **5** received in organic and in aqueous solution, respectively is given in Supporting Information File 1 (Figure S2) to show no significant differences. Also, CD signals in the range of 3.0 to 4.0 ppm are not observable. That indicates complete CD-decomplexation of **5**. Subsequently, **5** was epoxidized in a Prilezhaev reaction with *m*-chloroperbenzoic acid (*m*CPBA) in methylene chloride to obtain *N*,*N'*-(hexane-1,6-diyl)bis(4-methyl-*N*-(oxiran-2-ylmethyl)benzenesulfonamide) (**6**). The proceeding reaction was monitored by means of ^1^H NMR spectroscopy, since the allyl protons of **5** in the range of 5.0 to 5.7 ppm vanish on conversion (Figure S3, Supporting Information File 1). To illustrate the curing properties of the synthesized diepoxide, **6** was reacted in a ring-opening polymerization with the primary amine α-amino-ε-caprolactam (**8**). **8** was synthesized by cyclization of lysine (**7**) (Scheme 2). Hence, an increase of the reactivity of the primary amino group towards the epoxide function compared to the amino groups in native lysine was expected [26–28].

**Figure 2:**
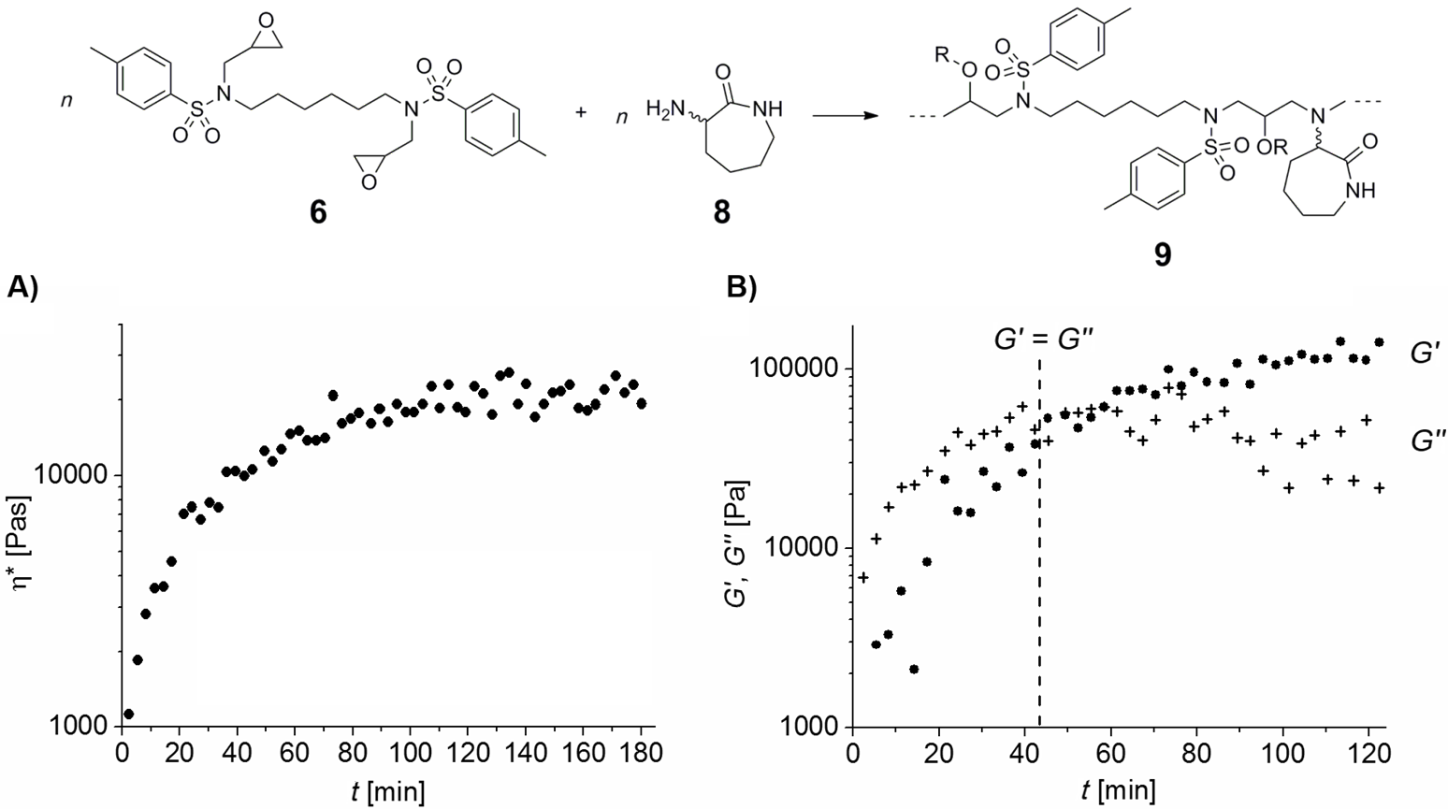
Oscillatory rheological measurements of an equimolar mixture of **6** and **8** at 50 °C. Illustrated is the complex viscosity (A), as well as *G’* and *G’’* (B) in dependence of time. The polymer **9** is displayed as an idealized structure (R = H or polymer network; crosslinking via addition of epoxide moieties to the hydroxy functions is likely [2]).

As illustrated in Figure 2A, oscillatory measurements display an initial high viscosity for a mixture of **6** and **8**, since its complex viscosity (η*), calculated from storage modulus *G’* and loss modulus *G’’*, exhibits low four-digit values at 50 °C. However, η* increases further on time until the sol–gel-transition (gelpoint), where *G’* is equal to *G’’*, is reached after about 40 to 45 min (Figure 2B). Generally, by passing this point the mixture is no longer capable of flowing. Shortly after reaching the gelpoint, η* is not significantly rising further, which is a sign for completed conversion. The resulting poly-adduct **9** exhibits a glass transition temperature (*T*_g_) of around 49 °C. Equivalent measurements of standard BADGE demonstrated a gelpoint after about 99 min and a *T*_g_ of about 74 °C (Figure S4, Supporting Information File 1). Thus, the curing properties for a mixture of **6** with **8** are relatively comparable to a mixture of BADGE with **8**, whereby the observed differences can be caused by different reactivity of the oxirane moieties or unequal solubility of the respective diepoxide with **8**.

By means of IR spectroscopy, ring opening polymerization of epoxides with amines can be monitored. The spectrum of **6** exhibits weak bands at 1253 and 895 cm^−1^, which can be assigned to the C–O-stretching vibration and the symmetric ring deformation vibration, respectively, of its epoxide groups. On curing at 50 °C, a broad band between 3100 and 3600 cm^−1^ appears which is caused by hydrogen bonded hydroxy stretching vibrations originating from epoxide ring opening. The epoxide bands seem to vanish after curing, which is a sign for high conversion. However, due to overlaps of broader bands in adjacent areas, no clear statement can be made in this regard (Figure S5, Supporting Information File 1). Based on this work, future research could focus on conducting the entire synthesis of **6** CD mediated in aqueous solution. Also, the presented pathway for alkylation in aqueous media could be transferred to the solvent saving synthesis of further alkylated products.

## Conclusion

An alternative route for the synthesis of *N*,*N'*-(hexane-1,6-diyl)bis(4-methyl-*N*-(oxiran-2-ylmethyl)benzenesulfonamide) (**6**) via *N*-alkylation of *N*,*N'*-(hexane-1,6-diyl)bis(4-methylbenzenesulfonamide) (**3**) and further oxidation was described. For the first time, the CD-mediated *N*-alkylation of a sulfonamide was conducted, whereby water solubility of **3** and **4** were increased by adding β-CD and α-CD, respectively. By applying oscillatory rheological measurements, a mixture of **6** and **8** reached the gelpoint after about 40 to 45 min at 50 °C. Thus, an environmentally more sustainable route for the synthesis of a bisphenol A free diepoxide was presented, which appears to be a suitable substitute for technically employed bisphenol A diglycidyl ether.

## Experimental

All reactants were commercially available and unless otherwise stated used without further purification. All solvents used were of analytical purity or freshly distilled. β-CD and α-CD were obtained from Wacker Chemie GmbH and were used after being dried with a vacuum oil pump over P_4_O_10_. *p*-Toluenesulfonyl chloride (98%) was obtained from Alfa Aesar, allyl bromide (99%), 3-chloroperoxybenzoic acid (70–75%), 1,6-hexanediamine (99.5+%) and L-(+)-lysine monohydrochloride (99+%) were purchased from Acros Organics, bisphenol A diglycidyl ether and deuterium oxide (99.9%) were provided from Sigma Aldrich, chloroform-*d* (99.80%) was obtained from Euriso-Top and dimethyl sulfoxide-*d*_6_ (99.9%) was purchased from Deutero. ^1^H NMR, ^13^C NMR and 2D ROESY spectra were recorded on a Bruker Avance DRX 300 and a Bruker Avance III – 600 by using deuterium oxide, dimethyl sulfoxide-*d*_6_ or chloroform-*d* as solvents. The chemical shifts (δ) are given in parts per million (ppm) using the solvent peak as an internal standard. FTIR spectra were recorded on a Nicolet 6700 FTIR spectrometer equipped with an ATR unit. Oscillatory rheological measurements were performed on a Haake Mars II rheometer by ThermoFisher Scientific. For this purpose a plate-plate construction (MP35, PP35Ti) was used. The temperature was set to 50 °C and was determined in the measuring plate with an accuracy of ±0.1 °C. The Sol–gel-transmission was determined by the first intersection of G’ and G’’. Graphs were plotted using reduced data, calculated by the median of three following values. The glass transition temperature (*T*_g_) was determined using a Mettler Toledo DSC 822e equipped with a sample robot TSO801RO. For calibration, standard tin, indium, and zinc samples were used. Heating and cooling curves were determined between −30 and 130 °C at a heating rate of 15 °C/min. The *T*_g_ value was taken from the inflection point of the second heating curve. Electrospray ionization mass spectrometry (ESIMS) was conducted on a Bruker maXis 4G mass spectrometer. Melting points were obtained using a Büchi Melting Point B-545 apparatus at a heating rate of 5 °C/min.

**Synthesis of 5 in organic solution:** 7.0 mmol of **3** were dissolved in 25 mL dried *N*,*N*-dimethylformamide and the solution was cooled to 0 °C in an ice bath. The apparatus was set under constant nitrogen-flow and 17.2 mmol of sodium hydride were added. Afterwards, the suspension was stirred for 2 h and subsequently, 35 mmol of allyl bromide (**4**), which was filtrated through neutral aluminium oxide, was added dropwise. The resulting mixture was stirred at 50 °C for 18 h and after cooling to room temperature it was diluted with 40 mL of saturated solution of ammonium chloride. The aqueous phase was washed threefold with 40 mL of ethyl acetate, the organic phases were combined and the solvent was evaporated under reduced pressure. After drying, 5.3 mmol (76% yield, not optimized) of brown slurry were received. The raw product was purified by column chromatography (*n*-hexane/ethyl acetate 2:1) to give 3.98 mmol (57% yield, not optimized) of **5**. ^1^H NMR (300 MHz, DMSO-*d*_6_, δ) 7.68 (d, *J* = 8.3 Hz, 4H, Ar-H), 7.40 (d, *J* = 7.9 Hz, 4H, Ar-H), 5.60 (ddt, *J* = 6.3, 10.1, 17.1 Hz, 2H, H_2_C=C*H*-CH_2_-), 5.19 (dd, *J* = 17.0, 1.6 Hz, 2H, CH_2_, *trans*), 5.11 (dd, *J* = 10.1, 1.6 Hz, 2H, CH_2_, *cis*), 3.73 (d, 4H, *J* = 6.4 Hz, allyl-CH_2_-), 2.99 (t, *J* = 7.4 Hz, 4H, N-CH_2_-), 2.39 (s, 6H, Ar-CH_3_), 1.38 (m, 4H, -CH_2_-), 1.14 (m, 4H, -CH_2_-); ^13^C NMR (75 MHz, DMSO-*d*_6_, δ) 143.0 (2C, Ar(*C*)-CH_3_), 136.6 (2C, RO_2_S-(C)Ar), 133.5 (2C, R-*C*H=CH_2_), 129.8 (4C, Ar(C)), 126.9 (4C, Ar(C)), 118.5 (2C, RHC=*C*H_2_), 50.1 (2C, N-*C*H_2_-CHR), 47.0 (2C, N-*C*H_2_-CH_2_R), 27.5 (2C, -CH_2_-), 25.5 (2C, -CH_2_-), 20.9 (2C, -CH_3_); IR (diamond) ν (cm^−1^): 2951, 2916, 2855 (m, -CH_2_-, Ar-CH_3_), 1652 (w, C=C), 1597 (m, Ar), 1336, 1155 (v, R-SO_2_-NR_2_), 1090 (m, Ar-S-), 965 (v, -CH_2_-), 934 (m, R-SO_2_-NR_2_), 816 (s, Ar-H (neighbouring)); ESIMS *m*/*z*: 505.4 [M + H]^+^, 527 [M + Na]^+^; mp 70 °C.

**Synthesis of 5 CD mediated in aqueous solution:** 6.8 g of randomly methylated β-CD and 25 mmol of sodium hydroxide were dissolved in 15 mL double distilled water and 2.4 mmol of **3** were added. The initial suspension was stirred at 45 °C for some minutes until a homogenous solution was achieved. Simultaneously, a homogenous solution of 1.15 mmol of **4**, 13.8 mmol of sodium hydroxide and 1.6 mmol of α-CD in 10 mL of double distilled water was prepared. Subsequently, the solution of **4α** was added dropwise to the solution of **3β**. The combined solutions were stirred at 50 °C and over the next 15 h further 8 mmol of **4** were added successively. After two days, the solution was heated to 70 °C for 30 min, filtrated and the precipitate dried in vacuo. 0.9 mmol (38% yield, not optimized) of a colorless powder of **5** was obtained. Spectral data of **5** synthesized in aqueous solution are given in the Supporting Information File 1.

**Synthesis of 6:** 3.0 mmol of **5** were dissolved in 15 mL of methylene chloride. Subsequently, 12.6 mmol of *m*CPBA dissolved in 20 mL methylene chloride were added. After two days of stirring at room temperature the reaction batch was treated with further 8.7 mmol of *m*CPBA and stirred for 4 days. Afterwards, the suspension was filtrated, the filtrate diluted with 20 mL of methylene chloride and washed three times with an aqueous solution of 10% sodium bisulfite. Then, the organic phase was treated twice with 50 mL each of sodium hydrogen carbonate solution and stirred for 1.5 h. Subsequently, the organic phase was dried over magnesium sulfate and the solvent removed under reduced pressure to obtain 1.7 mmol (57% yield, not optimized) of **6**. ^1^H NMR (300 MHz, CDCl_3_, δ): 7.67 (d, *J* = 8.3 Hz, 4H, Ar-H), 7.28 (d, *J* = 7.9 Hz, 4H, Ar-H), 3.59 (dd, *J* = 3.4, 15.2 Hz, 2H, N-C*H*_2_-CH-, *trans*), 3.15 (m, 4H, N-C*H*_2_-CH_2_-), 3.05 (m, 2H, -CH-), 2.87 (dd, *J* = 6.5, 15.2 Hz, 2H, N-C*H*_2_-CH-, *cis*), 2.74 (dd, *J* = 5.3, 4.2 Hz, 2H, O–CH_2_, *cis*), 2.49 (dd, *J* = 2.6, 4.3 Hz, 2H, O–CH_2_, *trans*), 2.40 (s, 6H, Ar-CH_3_), 1.55 (m, 4H, -CH_2_-), 1.27 (m, 4H, -CH_2_-); ^13^C NMR (75 MHz, CDCl_3_, δ) 143.6 (2C, Ar(*C*)-CH_3_), 136.8 (2C, RO_2_S-(C)Ar), 130.0 (4C, Ar(C)), 127.3 (4C, Ar(C)), 51.2 (2C, epoxy), 51.0 (2C, N-*C*H_2_-CH_2_R), 49.4 (2C, N-*C*H_2_-epoxy), 45.4 (2C, epoxy), 28.5 (2C, -CH_2_-), 26.3 (2C, -CH_2_-), 21.6 (2C, -CH_3_); IR (diamond) ν (cm^−1^): 2997, 2927, 2862 (m, -CH_2_-, Ar-CH_3_), 1597 (m, Ar), 1435 (m, -CH_2_-), 1335 (v, R-SO_2_-NR_2_), 1253 (w, -C-O-C-), 1154 (v, R-SO_2_-NR_2_), 1090 (m, Ar-S-), 895 (w, -C-O-C-), 839 (m, C-C (ring)), 816 (s, Ar-H (neighbouring)); ESIMS *m*/*z*: 537.1 [M + H]^+^, 559.1 [M + Na]^+^.

**Synthesis of 3 and 8:** Sulfonamide **3** was obtained in a modified synthesis similar to that reported in [23]. The synthesis of **8** was performed according to [29].

## Supporting Information

Experimental procedures and spectral data for the synthesis of **3** and **8** are given in the Supporting Information.

File 1 Additional spectra and experimental data.

## References

[1] Klee J E, Flammersheim H-J (2002). Macromol Chem Phys.

[2] Klee J, Hörhold H-H, Salamone J C (1996). Epoxide-amine addition polymers, linear. Polymeric Materials Encyclopedia.

[3] Wang L, Wu Y, Zhang W, Kannan K (2012). Environ Sci Technol.

[4] Resende L M, Rached-Junior F J A, Versiani M A, Souza-Gabriel A E, Miranda C E S, Silva-Sousa Y T C, Sousa Neto M D (2009). Int Endodont J.

[5] Fenouillot F, Rousseau A, Colomines G, Saint-Loup R, Pascault J-P (2010). Prog Polym Sci.

[6] Chiu Y-C, Chou I C, Tseng W-C, Ma C-C M (2008). Polym Degrad Stab.

[7] Shikha D, Kamani P K, Shukla M C (2003). Prog Org Coat.

[8] Tao Z, Yang S, Chen J, Fan L (2007). Eur Polym J.

[9] Pews R G (2006). Diepoxide derivatives of N,N*-disubstituted disulfonamides. U.S. Patent.

[10] Kolman A, Chovanec M, Osterman-Golkar S (2002). Mutat Res.

[11] Braun D, Lee D W (1976). Angew Makromol Chem.

[12] Braun D, Lee D W (1977). Angew Makromol Chem.

[13] Prileschajew N (1909). Ber Dtsch Chem Ges.

[14] Woods K W, Beak P (1991). J Am Chem Soc.

[15] Mimoun H (1982). Angew Chem, Int Ed Engl.

[16] Ritter H, Tabatabai M (2002). Prog Polym Sci.

[17] Alupei V, Alupei I C, Ritter H (2005). Macromol Rapid Commun.

[18] Wenz G (1994). Angew Chem, Int Ed Engl.

[19] Surendra K, Krishnaveni N S, Sridhar R, Rao K R (2006). Tetrahedron Lett.

[20] Zhang Q, Cheng G, Huang Y-Z, Qu G-R, Niu H-Y, Guo H-M (2012). Tetrahedron.

[21] Yamaguchi I, Osakada K, Yamamoto T (1997). Macromolecules.

[22] Li W, Zhang W, Ma X, Wang P, Du M (2012). Appl Catal, A: Gen.

[23] Stetter H, Roos E-E (1954). Chem Ber.

[24] Müller A, Sauerwald A (1927). Monatsh Chem.

[25] Tewari Y B, Miller M M, Wasik S P, Martire D E (1982). J Chem Eng Data.

[26] Philippe C, Milcent T, Crousse B, Bonnet-Delpon D (2009). Org Biomol Chem.

[27] Brotzel F, Chu Y C, Mayr H (2007). J Org Chem.

[28] Brotzel F, Mayr H (2007). Org Biomol Chem.

[29] Frost J W (2005). Synthesis of Caprolactam from Lysine. WO Patent.

