# Supplementary material for: N,N'-(Hexane-1,6-diyl)bis(4-methyl-N-(oxiran-2-ylmethyl)benzenesulfonamide): Synthesis via cyclodextrin mediated N-alkylation in aqueous solution and further Prilezhaev epoxidation
Source: Beilstein J Org Chem. 2013 Dec 9;9:2834–40. doi: 10.3762/bjoc.9.318 (PMC3869289; doi:10.3762/bjoc.9.318)
Supplement: File 1 — Additional spectra and experimental data. [file Beilstein_J_Org_Chem-09-2834-s001.pdf]

Supporting Information

for

N,*N'*-(Hexane-1,6-diyl)bis(4-methyl-*N*-(oxiran-2-ylmethyl)benzenesulfonamide): Synthesis via cyclodextrin mediated *N*-alkylation in aqueous solution and further Prilezhaev epoxidation

Julian Fischer, Simon Millan and Helmut Ritter*

Address: [1]Institute of Organic Chemistry and Macromolecular Chemistry, Heinrich-Heine-University Düsseldorf, Universitätsstraße 1, 40225 Düsseldorf, Germany

Email: Helmut Ritter – h.ritter@hhu.de
* Corresponding author

Additional spectra and experimental data

Content:

- Synthesis and spectral data of **3**
- Spectral data of **5**, synthesized CD mediated in aqueous solution
- Synthesis and spectral data of **8**
- 2D NMR ROESY of **4** with α-CD
- ^{1}H NMR spectrum of **5**
- ^{1}H NMR spectrum of **6**
- Oscillatory rheological measurement of BADGE with **8** at 50 °C
- IR spectra of **6**, **8** and a mixture of **6** and **8** after curing
- Reference

Synthesis of 3

62.9 mmol of *p*-toluenesulfonyl chloride (**1**) were dispersed in 17 mL of diethyl ether and cooled with an ice bath. A solution of 63.8 mmol of sodium hydroxide and 31.4 mmol of hexamethylenediamine (**2**) in 15 mL of double distilled water was added dropwise. Afterwards, the suspension was stirred thoroughly for 24 h at room temperature. The product was filtered off and recrystallized in 355 mL methanol. After drying in vacuo, 22.4 mmol (71% yield) of colorless crystals of **3** were obtained.

^{1}H NMR (600 MHz, DMSO-d_6, δ [ppm]): 8.17 (m, 4H, Ar-**H**), 7.84 (m, 4H, Ar-**H**), 6.76 (t, 2H, N-**H**), 3.29 (m, 4H, N-C$\mathbf{H}_2$), 2.86 (s, 6H, Ar-C$\mathbf{H}_3$), 1.86 (m, 4H, C$\mathbf{H}_2$), 1.67 (m, 4H, C$\mathbf{H}_2$); ^{13}C NMR (150 MHz, DMSO-d_6, δ [ppm]): 153.3 (2C, Ar-**C**H_3), 148.9 (2C, RO_2S-(**C**)-Ar), 140.0 (4C, **C**H, Ar), 137.4 (4C, **C**H, Ar), 53.4 (2C, N-**C**H_2), 36.4 (4C, **C**H_2), 31.0 (2C, Ar-**C**H_3); IR (diamond): ν (cm^{-1}) = 3267 (br, -NH-), 2943, 2883, 2859 (m, -CH_2-, Ar-CH_3), 1597 (m, C-C, Ar), 1417 (m, R_2-N-CH_2-), 1312, 1148 (v, R-SO_2-NH-), 1093 (m, Ar-S-), 938 (m, R-SO_2-NH-), 813 (s, Ar-H (neighbouring)); MS-ESI, *m/z*: 425.4 $[M + H]^+$; mp: 153 °C.

Spectral data for the CD mediated synthesis of 5 in aqueous solution

^{1}H NMR (300 MHz, DMSO-d_6, δ [ppm]): 7.68 (d, 4H, J = 8.3 Hz, Ar-H), 7.41 (d, 4H, J = 7.9 Hz, Ar-H), 5.60 (ddt, 2H, J = 6.3, 10.1, 17.1 Hz, H_2C=C**H**-CH_2-), 5.21 (dd, 2H, J = 17.2, 1.7 Hz, CH_2,*trans*), 5.11 (dd, 2H, J = 10.1, 1.6 Hz, CH_2,*cis*), 3.73 (d, 4H, J = 6.3 Hz, Allyl-CH_2-), 2.99 (t, 4H, J = 7.4 Hz, N-CH_2-), 2.39 (s, 6H, Ar-CH_3), 1.38 (m, 4H, -CH_2-), 1.14 (m, 4H, -CH_2-); ^{13}C NMR (75 MHz, DMSO-d_6, δ [ppm]): 143.0 (2C, Ar(**C**)-CH_3), 136.6 (2C, RO_2S-(C)Ar), 133.5 (2C, R-**C**H=CH_2), 129.8 (4C, Ar(C)),

126.9 (4C, Ar(C)), 118.5 (2C, RHC=**C**H_2), 50.1 (2C, N-**C**H_2-CHR), 47.1 (2C, N-**C**H_2-CH_2R), 27.5 (2C, -CH_2-), 25.5 (2C, -CH_2-), 20.9 (2C, -CH_3); IR (diamond): ν (cm^{-1}) = 2951, 2918, 2855 (m, -CH_2-, Ar-CH_3), 1652 (w, C=C), 1597 (m, Ar), 1336, 1155 (v, R-SO_2-NR_2), 1089 (m, Ar-S-), 964 (v, -CH_2-), 933 (m, R-SO_2-NR_2), 815 (s, Ar-H (neighbouring)); MS-ESI, *m/z*: 505.4 $[M+H]^+$, 527 $[M+Na]^+$; mp: 70 °C.

Synthesis of 8 [1]

A mixture of L-(+)-lysine monohydrochloride (**7**, 20 g, 109.5 mmol), sodium hydroxide (4.38 g, 109.5 mmol), aluminium oxide (60 g, 589 mmol) and 300 mL *n*-butanol were heated to reflux for 48 h in a reaction vessel equipped with a water trap. Subsequently, the mixture was filtrated and the obtained pale yellow solution was concentrated under reduced pressure. After precipitation in diethyl ether, filtration, and drying in vacuo 8.1 g (63.2 mmol = 57.7%) of colorless / pale yellow crystals were received.

^{1}H NMR (300 MHz, D_2O, δ [ppm]): 3.73 (dd, *J* = 11.0, 1.9 Hz, 1H, CH), 3.26 (m, 2H, RHN-CH_2-R), 2.02 – 1.30 (m, 6H, $(CH_2)_3$); ^{13}C NMR (75 MHz, D_2O, δ [ppm]): 181.3 (1C, C(O)), 52.7 (1C, CH), 41.3 (1C, RHN-CH_2-R), 32.4 (1C, RHC-CH_2-R), 27.7 (1C, CH_2), 27.6 (1C, CH_2); IR (diamond): ν (cm^{-1}) = 3355, 3283 (br, NH), 2930, 2909, 2849 (m, CH, CH_2), 1648 (s, amide); MS-ESI, *m/z*: 129.1 $[M+H]^+$; mp: 65–69 °C;

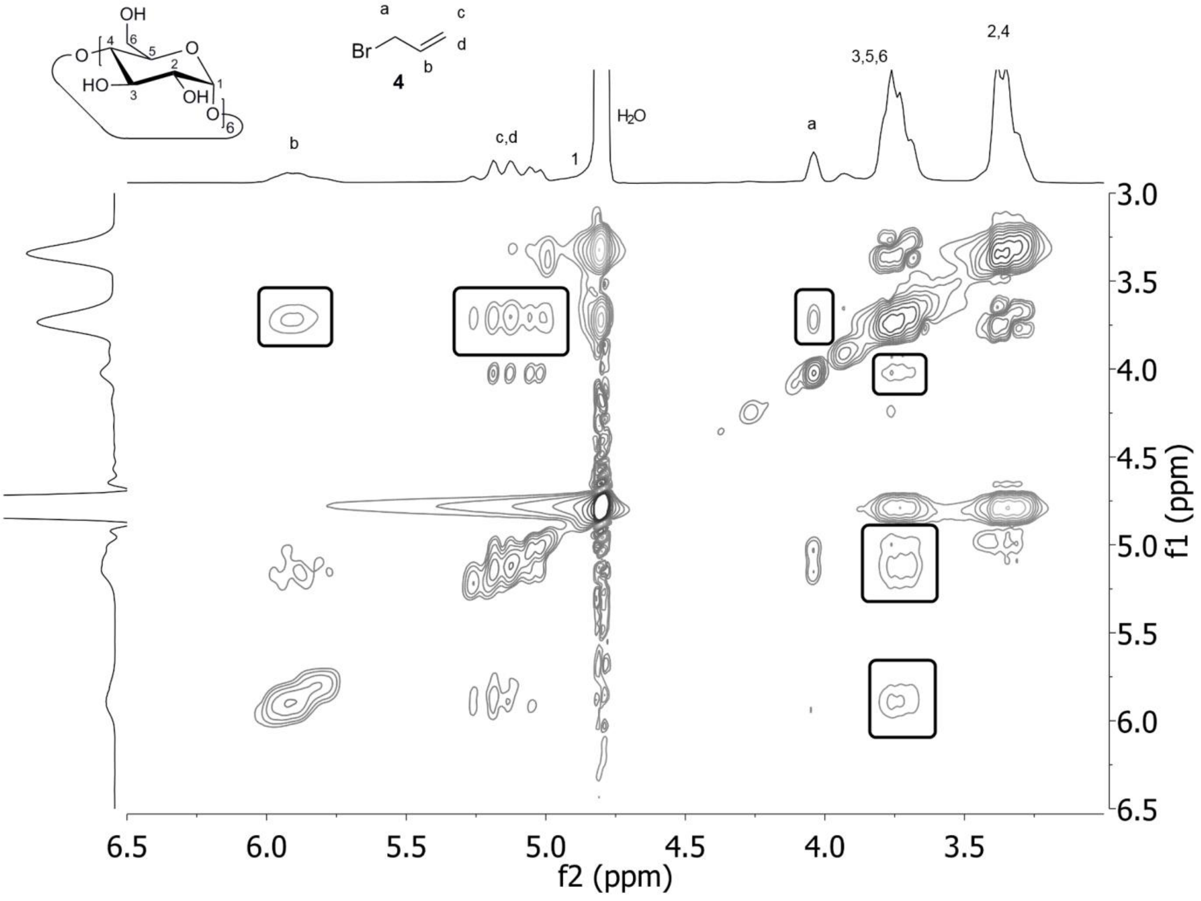

Figure S1: 2D NMR ROESY of the complex of **4** with α-CD in D_2O.

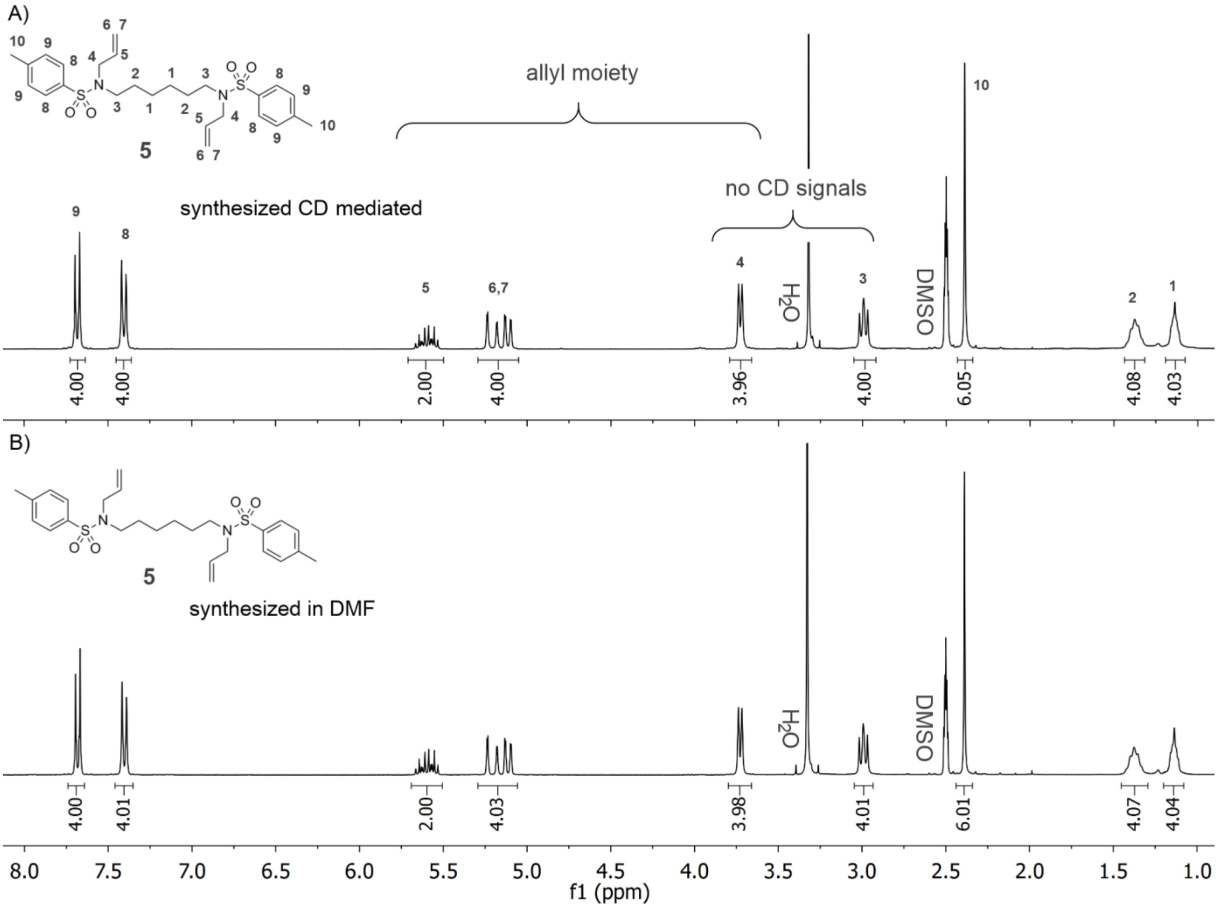

Figure S2: ^{1}H NMR spectra (DMSO-d_6) of the dialkylated sulfonamide **5**, synthesized CD mediated in aqueous solution (A) and in organic solution (*N*,*N*-dimethylformamide = DMF) (B).

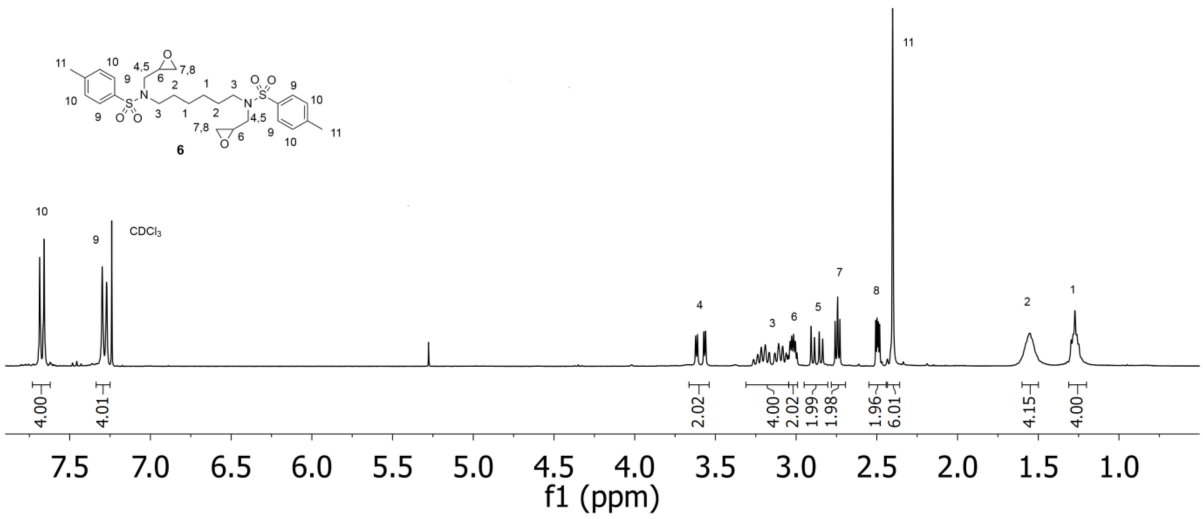

Figure S3: ^{1}H NMR spectrum of **6** in $CDCl_3$.

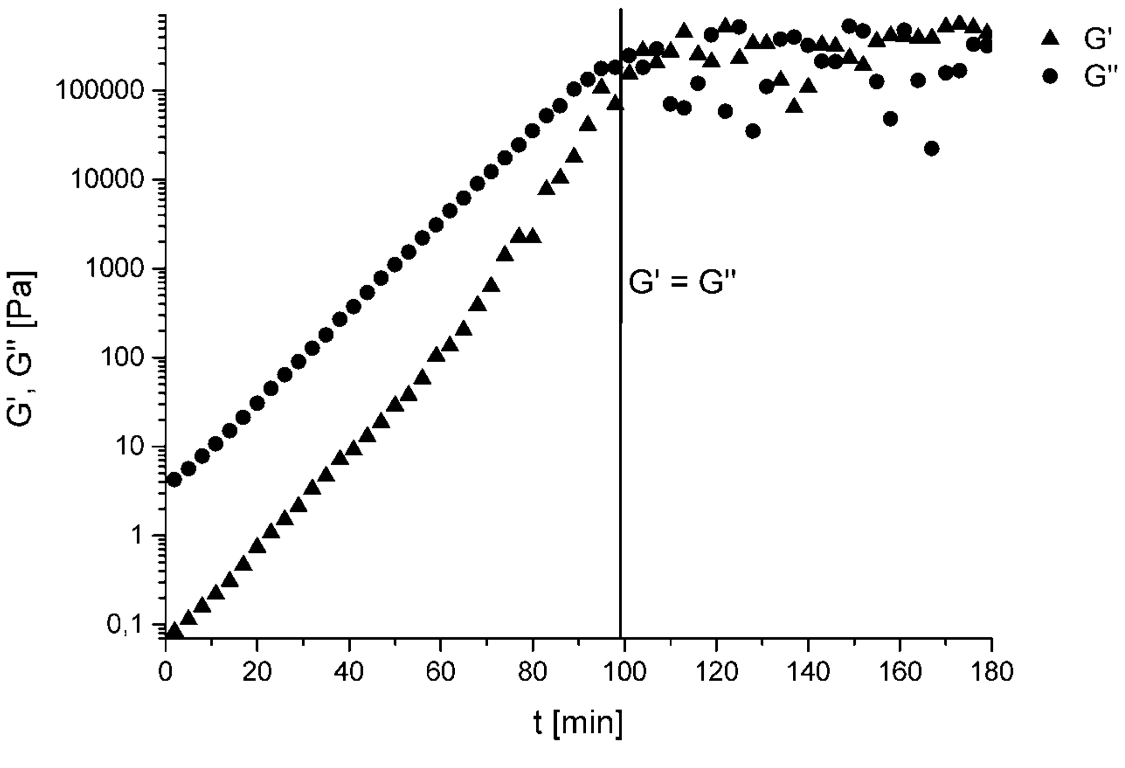

Figure S4: Oscillatory rheological measurement of BADGE with **8** at 50 °C.

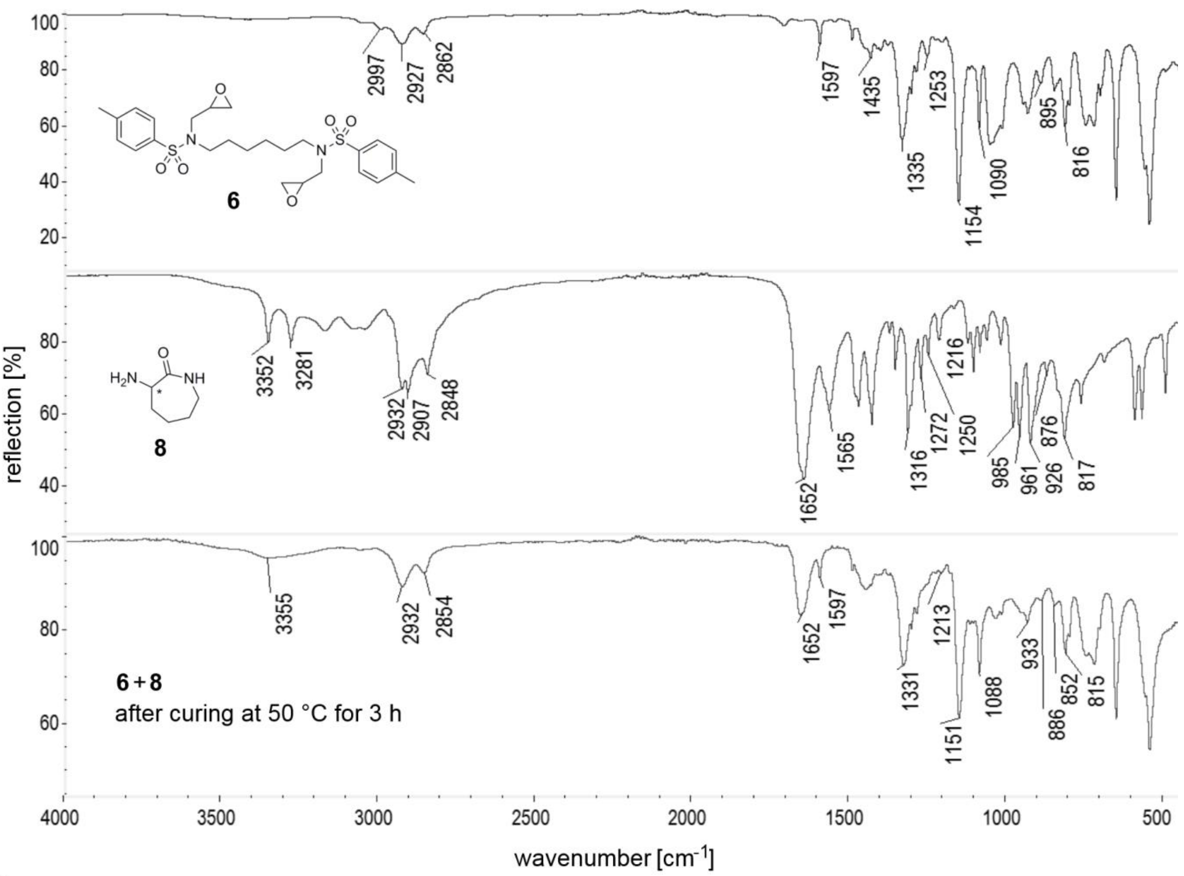

Figure S5: IR spectra of **6**, of **8**, and of a mixture of **6** and **8** after curing at 50 °C for 3 h.

Reference

1. Frost, J. W.; Board of trustees of Michigan State University. Synthesis of Caprolactam from Lysine. WO Patent 123669A1, December 29, 2005.
